# Human-immunodeficiency virus infection associated with the impaired Th1 and pro-inflammatory cytokine response in latent tuberculosis-infected individuals: A comparative cross-sectional study

**DOI:** 10.1371/journal.pone.0313306

**Published:** 2024-11-08

**Authors:** Getu Girmay, Amare Kiflie, Meseret Alem, Mulualem Lemma, Gezahegn Bewket

**Affiliations:** Department of Immunology and Molecular Biology, School of Biomedical and Laboratory Science, College of Medicine and Health Sciences, University of Gondar, Gondar, Ethiopia; ICMR-National Institute for Research in Tuberculosis, INDIA

## Abstract

Tuberculosis (TB) and HIV co-infections are extensively overlapping, especially in developing countries. HIV infection is known as a major risk factor for the reactivation of latent TB into active TB. Although not fully understood and needs further study, HIV infection might enhance the reactivation of latent TB by breaching immune control mechanisms. We investigated the influence of HIV infection on the cytokine response of LTB-infected individuals. Heparinized venous blood was collected from 40 ART-naïve HIV-infected and 30 HIV-negative healthy controls for LTB screening, plasma collection, and PBMC isolation and stimulation. The level of cytokines in plasma and their production by PBMCs stimulated with purified protein derivative (PPD), staphylococcus enterotoxin B (SEB), or unstimulated PBMCs were analyzed using a cytometric bead array (CBA) assay. PPD-induced IL-2 by PBMCs was higher in LTB-infected groups compared with HIV-negative LTB-negative groups (p = 0.0015). When LTB-infected groups were co-infected with HIV (HIV^+^LTB^+^), the IL-2 (p < 0.0001) and IFN-gamma (p = 0.0144) production by PPD-stimulated PBMCs was reduced. The level of IL-2 (p = 0.0070), IL-6 (p = 0.0054), and TNF-alpha (p = 0.0045) in plasma were lower in HIV^+^LTB^+^ individuals compared with HIV-negative LTB-positive (HIV^-^LTB^+^) groups. Our findings suggested that HIV co-infection in LTB-positive individuals is associated with the diminished production of PPD-induced Th1 (IFN-gamma and IL-2) cytokines by PBMCs and in the plasma level of IL-2 and proinflammatory cytokines (IL-6 and TNF-alpha).

## Introduction

Nearly one-fourth (23.0%) of the world population is infected with latent tuberculosis (LTB) [1]. In 2022, an estimated 10.6 million new TB cases and 1.3 million deaths were reported, of whom approximately 8% of TB-associated deaths were people living with human immunodeficiency virus (HIV) [2, 3]. Ethiopia is grouped as one of the 30 countries with the highest burdens of TB-HIV co-infections [4]. HIV infection fuels tuberculosis epidemics by increasing the reactivation rate of latent tuberculosis infection (LTBI) to active tuberculosis (ATB) [5, 6]. Approximately 5 to 10% of LTBI individuals develop active TB by reactivation later in life, and the majority of them do so within 2 to 5 years of infection [7]. However, if LTBI individuals are co-infected with HIV, their risk of reactivation increases by 10% each year [6, 7].

During LTBI, the immune response is mainly cell-mediated, in which Mtb antigen-specific T cells provoke granuloma formation at the site of infection [8]. The granuloma consists of immune cells such as macrophages, CD4+ T cells, CD8+ T cells, regulatory T cells, and natural killer T (NKT) cells, which help to contain Mtb and keep the infected individuals latent. However, the granuloma also allows for the long-term survival of Mtb, which might be disseminated and cause active TB, especially when there are conditions that can compromise an individual’s immunity [9–11] such as HIV infection. Cytokines are pivotal in mediating the communication of various immune cells involved in granuloma formation and maintaining tuberculosis granuloma structure [12, 13]. It has been shown that the number of CD4+ T cells, the main producers of cytokines, decreased in LTBI individuals with HIV co-infection. In addition, the degree of the initial CD4+T cell depletion and their associated cytokines were linked with the likelihood of LTB reactivation in a monkey with Mtb and simian immunodeficiency virus 4 (SIV-4) co-infection [14, 15].

Th1 cytokines such as IFN-gamma and IL-2 are pivotal in immunity to Mtb infections [16]. IFN-gamma enhances the transcription of more than 200 genes in macrophages and activates them to produce reactive oxygen species and nitric oxide that keep the intact structure of Mtb-containing granuloma [17, 18]. IL-2 also participates in the cell-mediated immune response during Mtb infection by enhancing T-cell survival and proliferation [19]. Pro-inflammatory cytokines such as TNF-alpha and IL-6 enhance macrophages’ ability to phagocytose and combat Mtb and increase the synthesis of acute-phase proteins [20–22]. In addition, the tuberculin skin test-positive individuals were more vulnerable to LTB reactivation into ATB when they received antibody-dependent TNF-alpha blocking. The mutant mice deficient in IL-6 production were also extremely susceptible to tuberculosis disease progression [23, 24].

IL-17A has an essential role in TB immunity by enhancing the recruitment of phagocytic cells like neutrophils and maturing the nascent granuloma [25]. In IL-17A gene knockout mice, the recruitment of protective immune cells such as lymphocytes and neutrophils was reduced, and the development of granuloma structure failed [26, 27]. The Th2 cytokines, for instance, IL-4 and IL-10, have been associated with a defect in human macrophages to control intracellular Mtb and lead to an exacerbated TB disease progression [28, 29].

HIV infection has been associated with the impaired production of Th1 cytokines such as IFN-gamma and IL-2, which have a pivotal role in the immune response against Mtb [30]. The Mtb antigen (ESAT-6)-specific Th1 cells and their IFN-gamma responses were decreased in HIV-LTB co-infected individuals [31]. The decreased IFN-gamma production has been linked to the likelihood of LTB reactivation in the Cynomolgus macaques animal model [15]. The production of TNF-alpha in the early stages of granuloma formation in Simian immunodeficiency virus (SIV) and Mtb co-infected animals was reduced [32]. The level of IL-17A was also impaired in LTB-infected individuals with HIV co-infection [33]. Hence, the high burden of TB in areas where HIV infection is common might be linked with the HIV-mediated impaired production of essential cytokines, which maintain the immune control mechanisms during LTBI. Therefore, we aimed to investigate the impact of HIV infection on the production of Th1, pro-inflammatory, Th17, and Th2 cytokines in LTB-infected individuals in Gondar, Ethiopia, where TB-HIV co-infections are common.

## Materials and methods

### Ethical statement

The study received ethical approval from the ethics committee of the School of Biomedical and Laboratory Science, University of Gondar, Ethiopia, with a reference number of SBMLS/2754/2021. Written informed consent was obtained from all study participants.

### Study participants

**Fig 1:**
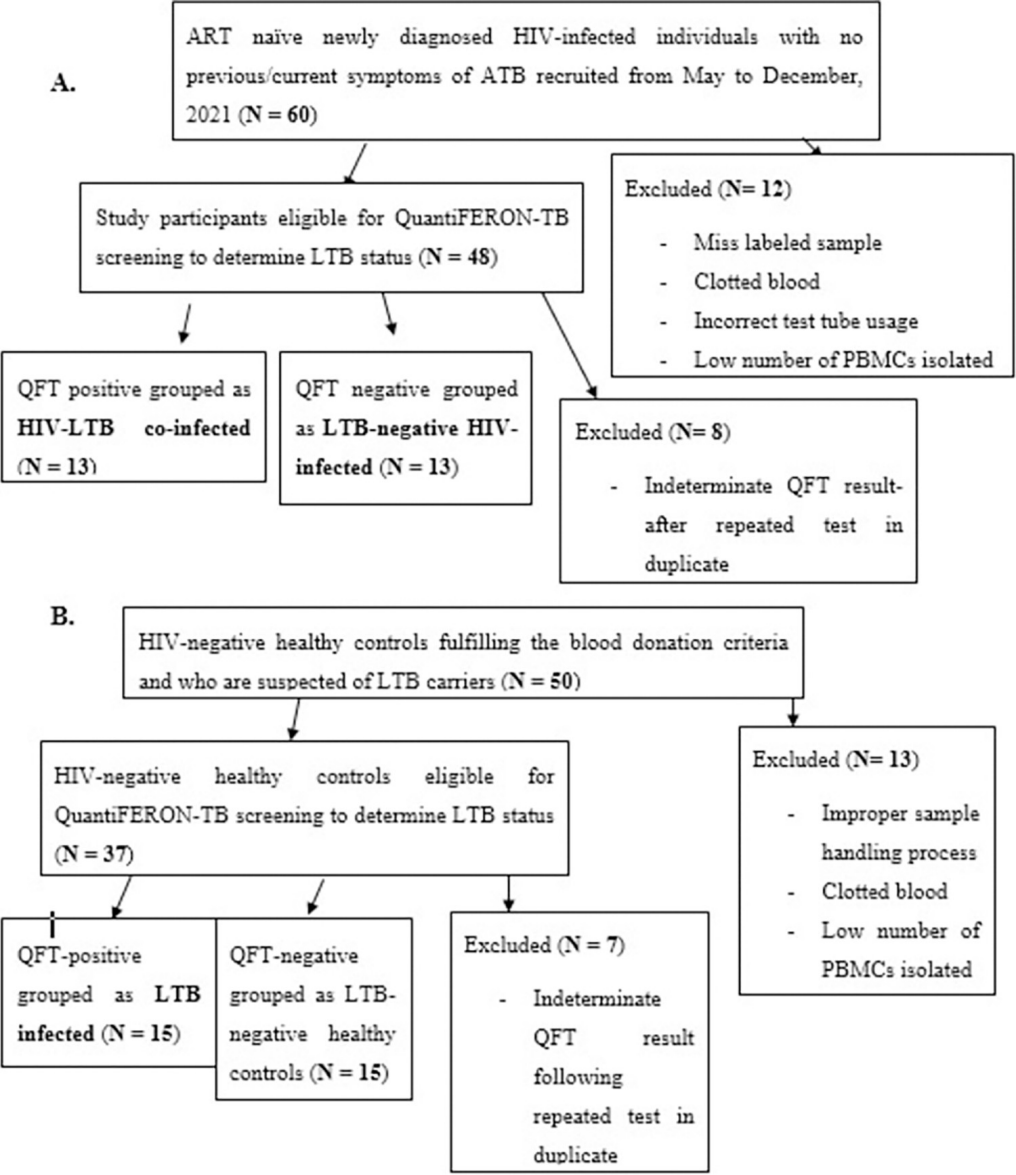
Flow chart of the recruitment and enrollment process of study participants. ART- naïve newly diagnosed HIV-infected individuals were recruited from the ART clinics of the UoG-CSH, Gondar Health Center, and Maraki Health Center, Gondar, Ethiopia (**A**). The HIV-negative healthy controls fulfilling the blood donation criteria were recruited from the central Gondar blood bank, Gondar, Ethiopia (**B**).

Newly diagnosed HIV-infected individuals were consecutively recruited from May 07, 2021, to December 15, 2021 at the antiretroviral therapy (ART) clinics of the University of Gondar Comprehensive Specialized Hospital (UoG-CSH), Gondar Health Center, and Maraki Health Center. The inclusion criteria were treatment naïve HIV-infected individuals with an age range of 18 to 65 years old, with confirmed HIV-positive status as part of a provider-initiated HIV counseling program and testing (**Fig 1A**). HIV-negative healthy controls fulfilling the blood donation criteria were recruited from the Central Gondar blood bank, Gondar, Ethiopia (**Fig 1B**). HIV-infected and-HIV negative individuals with a previous history or current active TB, BCG vaccination, helmenths infections, pregnancy, treatment with immunosuppressive drugs, diabetes mellitus, and any other acute or chronic medical conditions were excluded from the study.

The QuantiFERON-TB Gold Plus (QFT-plus) assay (Qiagen, Australia) test was performed according to the manufacturer’s instructions to define the LTBI status of the study participants [34]. Following the QFT-plus test, HIV-infected individuals were sub-grouped into HIV-infected LTB-negative (HIV^+^LTB^-^) and HIV-LTB co-infected (HIV^+^LTB^+^), and HIV-negative healthy controls were also grouped into HIV-negative LTB-negative (HIV^-^LTB^-^) and HIV-negative LTB-infected (HIV^-^LTB^+^) healthy controls.

### Socio-demographic and clinical data collection

Socio-demographic and clinical data were collected using a structured questionnaire by trained clinical nurses who worked in the selected ART clinics and Central Gondar blood bank service for HIV-infected individuals and HIV-negative healthy controls, respectively.

### HIV screening

The study participants in the selected ART clinics were screened for HIV infection as part of a provider-initiated HIV counseling program and testing and as a national HIV test algorism. HIV screening was done according to the clinic routine using HIV rapid test kits for HIV 1/2 STAT-PAK Assay (Chembio Diagnostics Systems Inc., USA), ABON™ HIV 1/2/O Tri-line assay (Abbon Biopharm, China), and SD BIOLINE HIV-1/2 3.0 (Standard Diagnostics, India) by strictly following the manufacturer’s instructions.

### CD4+ T cell count

CD4+ T cell count was done for HIV-positive study participants in the selected ART clinics using a BD FACS Presto^TM^ Near-Patient CD4 Counter (BD Bioscience, USA). The BD FACS Presto^TM^ cartridge contains dried fluorochrome-conjugated monoclonal antibodies (CD4 PE-CyTM5, CD3 APC, CD45RA APC, and CD14 PE). After adding the whole blood to the cartridges and incubating for 18 minutes, the conjugated antibodies specifically bind to the antigens on the surface of the cells. The dedicated software already installed in the FACS Presto^TM^, identified and enumerated the absolute count and percentage of CD4+ T cells when the stained cartridge was placed on the counter [35].

### QuantiFERON-TB Gold plus (QFT-plus) assay

Heparinized venous blood was collected from HIV-positive individuals and HIV-negative healthy controls. The QFT-plus assay (Qiagen, Australia) was used to measure the released IFN-gamma following in-vitro stimulation of whole blood with Mtb-specific early secretary antigen target-6 (ESAT-6) and culture-filtrated protein-10 (CFP-10) antigens. One milliliter (1ml) of blood was transferred to each of four tubes labeled (Nil, Mtb Ag-1, Mtb Ag-2, and Mitogen), and then incubated for 18 hours. The LTBI status was determined by measuring IFN-gamma from plasma samples harvested from QFT-plus tubes’ supernatants using an enzyme-linked immunosorbent assay (ELISA). The ELISA results were interpreted using the QuantiFERON TB Gold Plus (TBQFT) version 2.71.2 analysis software [34].

### Peripheral blood mononuclear cell (PBMC) isolation

Heparinized venous blood was collected from HIV-positive individuals and HIV-negative healthy controls and transported to the laboratory within two hours of collection for PBMC isolation. The PBMC isolation was performed as we did previously [36–38]. The heparinized whole blood (6 ml) was carefully layered on top of the LymphoPrep density gradient solution (Serumwerk, Bernburg AG, Oslo, Norway), and centrifuged at 800 g for 30 minutes at 20°C. Following centrifugation, the distinct PBMC layer formed above the LymphoPrep solution and underneath the plasma was harvested. Plasma was collected and stored at -80°C for later cytokine analysis. The PBMCs underwent two successive washings at 200 g for 10 minutes using a sterile phosphate-buffered saline (PBS) solution [39, 40].

### PBMCs counting, stimulation, and harvesting

PBMCs were resuspended in Roswell Park Memorial Institute (RPMI) 1640 medium (Sigma Aldrich, UK) supplemented with 10% sterile heat-inactivated Fetal Bovine Serum (FBS) (Invitrogen, USA) and a 1% Antibiotic-Antimycotic solution (Sigma Aldrich, UK). Then, PBMCs were counted in a Bürker counting chamber with 0.4% Trypan blue staining dye (Sigma-Aldrich, Munich, Germany). Cell stimulation and harvesting of the supernatant were performed as we did previously [37]. Half a million PBMCs were stimulated with Mtb-derived PPD (from Statens Serum Institute, Copenhagen, Denmark) at a final concentration of 10 μg/ml, stimulated with SEB (Sigma Aldrich, UK) at a final concentration of 5 μg/ml, and kept unstimulated (culture media only) as a background value and incubated for 19 hours at 37°C. After incubation, the PBMC supernatants were harvested using centrifugation at 800 g for 5 minutes and stored at -80°C for later extracellular cytokine analysis using a cytometric bead array (CBA) assay [40].

### Cytometric bead array (CBA) assay

Cytokine analysis was performed using the Human Th1/Th2/Th17 CBA kit (BD, Biosciences, USA) according to the manufacturer’s instructions. Seven cytokines (IL-2, IL-4, IL-6, IL-10, IL-17A, TNF-alpha, and IFN-gamma) can be measured simultaneously with this kit. The following is a description of the kit’s theoretical detection limit for each cytokine: IL-2 is 2.6 pg/ml, IL-4 is 4.9 pg/ml, IL-6 is 2.4 pg/ml, IL-10 is 4.5 pg/ml, IL-17A is 18.9 pg/ml, TNF-alpha is 3.8 pg/ml, and IFN-gamma is 3.7 pg/ml. Standard and unknown samples were mixed with beads coated with capture antibodies targeted to the specific cytokines and Phycoerythrin (PE) conjugated detection antibodies, and then incubated for 3 hours in the dark. Following incubation, samples were washed and analyzed using FACS Calibur flow cytometer (BD Biosciences, USA) using Cell Quest acquisition software. The intensity of the PE fluorescence of each sandwich complex reveals the concentration of specific cytokines [41]. The flow cytometer data were analyzed using FlowJo version 10.8.1 software (BD Biosciences, USA). The concentration of each cytokine was calculated with the help of a standard curve using Microsoft Excel from the median fluorescence intensity of each cytokine bead.

### Statistical analysis

The data were entered and analyzed using GraphPad Prism version 9.0 (GraphPad Software, Inc, La Jolla, CA, USA). Continuous variables were presented using median and interquartile range (IQR). The Kruskal-Wallis test was applied to show the median cytokine concentration difference between study groups, followed by Dunn’s post hoc test for multiple comparisons within each group. Correlation analysis was done using Spearman’s correlation. A p-value of < 0.05 was considered statistically significant.

## Results

### Socio-demographic and clinical characteristics of study participants

A total of 70 study participants: 27 HIV-infected LTB negatives (HIV^+^LTB^-^), 13 HIV-LTB co-infected (HIV^+^LTB^+^), 15 HIV-negative LTB-infected (HIV^-^LTB^+^), and 15 HIV-negative LTB-negative (HIV^-^LTB^-^) were included in this study. The median age with IQR was 32 (25–37), 34 (27–43), 28 (26–31), and 26 (24–28) years for HIV^+^LTB^-^, HIV^+^LTB^+^, HIV^-^LTB^+^, and HIV^-^LTB^-^, respectively. The distribution of female participants was lower in HIV^-^LTB^+^ groups compared with HIV^+^LTB^-^ groups (statistics performed by Fisher’s Exact test). The median age of HIV^+^LTB^+^ groups was higher than HIV^-^LTB^-^ groups. The BMI of HIV^+^LTB^-^ groups was lower compared with HIV^+^LTB^+^, HIV^-^LTB^+^, or HIV^-^LTB^-^ groups, respectively. However, after we applied Spearman’s correlation, age or BMI did not show a strong correlation with the concentration of each cytokine (**S1 Table**). There were no significant differences between HIV^+^LTB^-^ and HIV^+^LTB^+^ groups in terms of CD4+ T cell count and HIV clinical stage (**Table 1**).

**Table 1:** Socio-demographic and clinical characteristics of study participants.

Variables		HIV^+^LTB^-^	HIV^+^LTB^+^	HIV^-^LTB^+^	HIV^-^LTB^-^	p-value
No of cases		27	13	15	15	
Age^a^(year)		32 (25–37)	34 (27–43)	28 (26–31)	26 (24–28)	p^a^< 0.01
Sex^b^	Female	19(70.4%)	8 (61.5%)	2 (13.3%)	6 (40%)	P^d^<0.01
	Male	8 (29.6%)	5 (38.5%)	13 (86.7%)	9 (60%)	
BMI^a^ (Kg/m^2^)		19 (17–21)	21 (19.5–25)	22.6(19.1–25.67)	22.68 (19.92–23.33)	p^b^<0.05, P^c^<0.05, p^d^<0.05
HIV Clinical stage^b^	I	19 (70.4)	10 (76.9%)	NA	NA	NS
	II	6 (22.2)	2 (15.4%)			
	III	2 (7.4)	1 (7.7%)			
CD4+T cell count (cells/μl)^a^		190 (104.75–311.75)	245 (157–313)	ND	ND	NS

^**a**^: Median with interquartile range (IQR) for continuous variables

^**b**^: Frequency (%) for categorical variables. BMI: Body mass index; Kg/m^2^: kilogram per meter square; CD4: Cluster of Differentiation 4; NA: not applicable; ND: not done. Comparison of median age, BMI, and CD4+ T cells was performed using the Kruskal Wallis test followed by Dunn’s post hoc multiple comparisons test. The difference in sex distribution among study groups was analyzed by Fisher’s Exact test. Only significant p values are displayed. p^a^: p value of HIV^+^LTB^+^ versus HIV^-^LTB^-^; p^b^: p value of HIV^+^LTB^+^ versus HIV^+^LTB^-^; P^c^: p value of HIV^+^LTB^-^ versus HIV^-^LTB^-^; p^d^ p value of HIV^+^LTB^-^ versus HIV^-^LTB^+^. HIV^+^LTB^-^: HIV positive-LTB negative; HIV^+^LTB^+^: HIV-LTB co-infected; HIV^-^LTB^+^: HIV negative- LTB positive; HIV^-^LTB^-^: HIV-negative LTB-negative. NS: not statistically significant

*: p < 0.05, and

**: P < 0.01.

#### HIV infection associated with the decreased level of Th1 cytokines in latent TB-infected and LTB-negative individuals

To investigate whether HIV infection affects the Th1 immune response in LTB-infected individuals, we measured the concentration of IFN-gamma and IL-2 in plasma and from the PBMC supernatants with or without stimulation of PPD or SEB antigens.

The level of PPD-induced IFN-gamma (p = 0.0144) and IL-2 (p < 0.0001) production by PBMCs was significantly reduced in HIV^+^LTB^+^ groups compared with HIV^-^LTB^+^ groups. There was a significantly lower level of PPD-induced IFN-gamma (p < 0.0001) and IL-2 (p < 0.0001) production by PBMCs among HIV^+^LTB^-^ groups compared to HIV^-^LTB^+^ groups. The HIV^+^LTB^-^groups also had significantly decreased levels of IFN-gamma production in PPD-stimulated PBMCs (p = 0.0016) compared with HIV^-^LTB^-^ groups.

**Fig 2:**
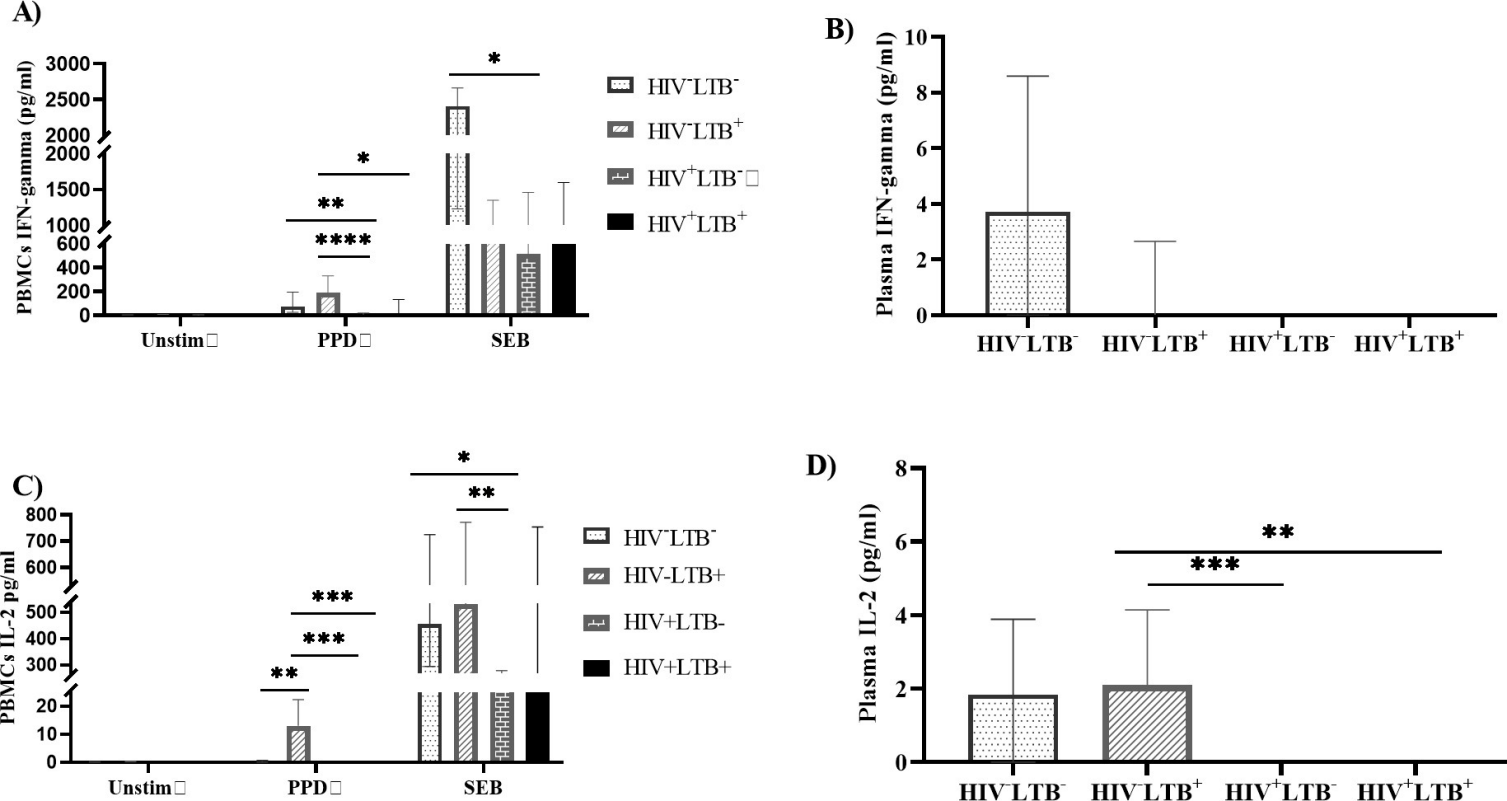
HIV infection significantly reduced the level of Th1 cytokines in LTB-infected and LTB-negative individuals. The PBMCs isolated from HIV^+^LTB^-^ (n = 27), HIV^+^LTB^+^ (n = 13), HIV^-^LTB^+^ (n = 15), and HIV^-^LTB^-^ (n = 15) were stimulated with media alone (Unstim), PPD or SEB and cultured for 19 hours at 37°C, and the PBMC supernatants were harvested. The levels of IFN-gamma and IL-2 were determined in plasma and from PBMC supernatants using a cytometric bead array. Data were presented using the median with IQR. The cytokine response was compared across the four groups using the Kruskal Wallis test, followed by Dunn’s post hoc multiple comparisons test to compare the median expression level of cytokine in each group. **A**): level of IFN-gamma production by PBMCs with or without stimulations, **B**): level of IFN-gamma in plasma, **C):** level of IL-2 production by PBMCs with or without stimulations, **D**): level of IL-2 in plasma. Unstim: unstimulated; PPD: purified protein derivative; SEB: staphylococcus enterotoxin B; HIV^+^LTB^-^: HIV positive-LTB negative; HIV^+^LTB^+^: HIV-LTB co-infected; HIV^-^LTB^+^: HIV negative- LTB positive; HIV^-^LTB^-^: HIV-negative and LTB-negative. *: p < 0.05, **: p < 0.01, ***: p < 0.001.

Although there was no significant difference in the plasma level of IFN-gamma in any of the groups, the level of IL-2 in plasma was significantly lower in HIV^+^LTB^+^ groups compared to HIV^-^LTB^+^ groups (p = 0.0070). Moreover, following SEB stimulation of PBMCs, we found a significantly lower level of IFN-gamma (p = 0.0019) and IL-2 (p = 0.0160) production in HIV^+^LTB^-^ groups compared with HIV^-^LTB^-^groups. The IL-2 production by SEB-stimulated PBMCs in HIV^+^LTB^-^groups was also significantly lower compared to HIV^-^LTB^+^ groups (p = 0.0016) **(Fig 2A–2D)**.

#### HIV infection caused a reduced plasma level of pro-inflammatory cytokines in LTB-infected LTB-negative individuals

**Fig 3:**
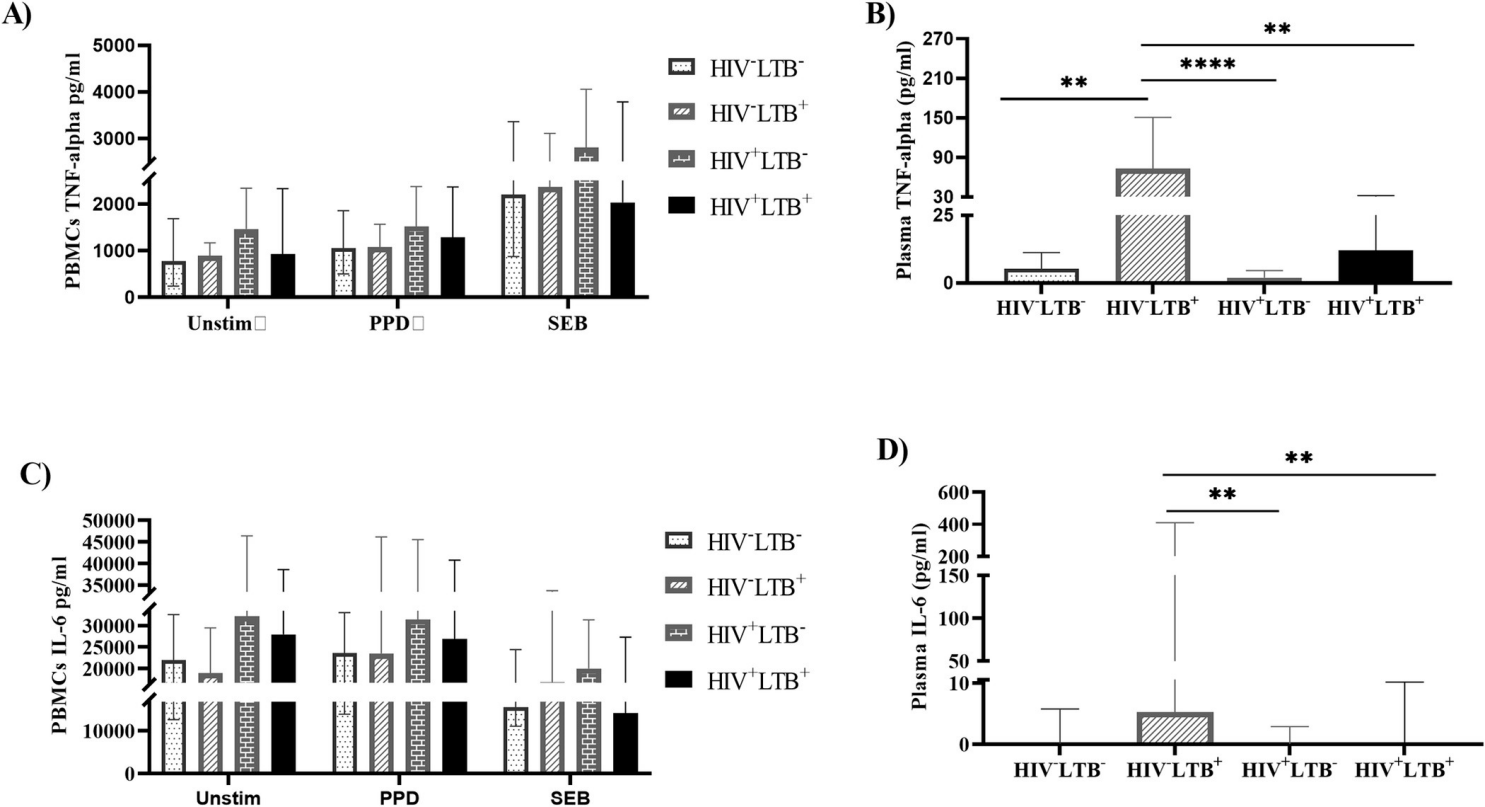
Reduced plasma level of pro-inflammatory cytokines in HIV-LTB co-infected and HIV positive LTB negative individuals. The PBMCs isolated from HIV^+^LTB^-^ (n = 27), HIV^+^LTB^+^ (n = 13), HIV^-^LTB^+^ (n = 15), and HIV^-^LTB^-^ (n = 15) individuals were stimulated with media alone (Unstim), PPD or SEB and cultured for 19 hours at 37°C, and PBMC supernatants were harvested. The levels of TNF-alpha and IL-6 were determined in plasma and from PBMC supernatants using a cytometric bead array. Data were presented using median with IQR. The cytokine response was compared across the four groups using the Kruskal-Wallis test, followed by Dunn’s post hoc multiple comparisons. **A**): levels of TNF-alpha production by PBMCs with or without stimulations, **B**): levels of TNF-alpha in plasma, **C)**:levels of IL-6 production by PBMCs with or without stimulations, **D):** levels of IL-6 in plasma; Unstim: unstimulated; PPD: purified protein derivative; SEB: staphylococcus enterotoxin B; HIV^+^LTB^-^: HIV positive-LTB negative; HIV^+^LTB^+^: HIV-LTB co-infected; HIV^-^LTB^+^: HIV negative- LTB positive; HIV^-^LTB^-^: HIV-negative and LTB-negative. *: p < 0.05, **: p < 0.01, ***: p < 0.001.

In our study, we analyzed pro-inflammatory cytokines (TNF-alpha and IL-6) in the harvested plasma and PBMC supernatants without stimulation and following stimulation with PPD and SEB antigens. The systemic level of TNF-alpha and IL-6 in plasma was significantly reduced in HIV^+^LTB^+^ groups compared with HIV^-^LTB^+^ groups (p = 0.0045 and p = 0.0054, respectively). Moreover, the plasma level of TNF-alpha was significantly lower in HIV^+^LTB^-^groups compared with HIV^-^LTB^-^ groups (p < 0.0001). The level of plasma IL-6 was also significantly lowered in HIV^+^LTB^-^ groups compared with HIV^-^LTB^-^groups (p = 0.0054). However, there was no significant difference in the production of TNF-alpha and IL-6 between any of the study groups from PBMC supernatants, both in unstimulated and following stimulation with PPD or SEB antigens (**Fig 3A–3D**).

#### HIV infection did not affect the production of IL-17A in LTB-infected and LTB negative individuals

**Fig 4:**
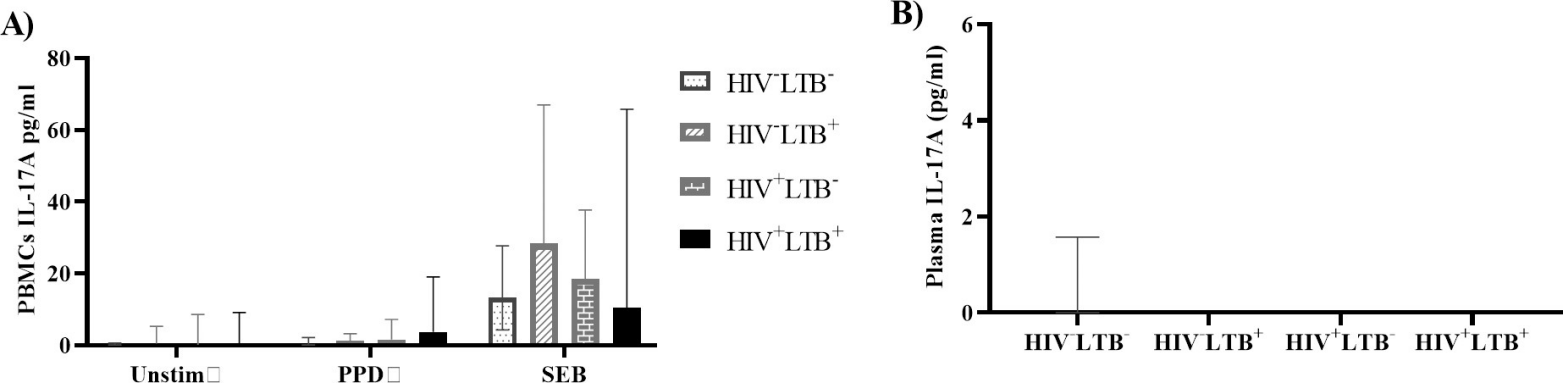
The level of IL-17A in HIV-LTB co-infected and LTB-infected individuals was not significantly different. PBMCs isolated from HIV^+^LTB^-^ (n = 27), HIV^+^LTB^+^ (n = 13), HIV^-^LTB^+^ (n = 15) and HIV^-^LTB^-^ (n = 15) were stimulated with media alone (Unstim), PPD or SEB and cultured for 19 hours at 37°C, and PBMC supernatants were harvested. The level of IL-17A was determined in plasma and from PBMC supernatants using a cytometric bead array. Data were presented in the median with IQR. The cytokine response was compared across the four groups using the Kruskal-Wallis test, followed by Dunn’s post hoc multiple comparisons. **A**): the level of IL-17A production by PBMCs with or without stimulation, **B):** level of IL-17A in plasma Unstim: unstimulated; PPD: purified protein derivative; SEB: staphylococcus enterotoxin B; HIV^+^LTB^-^: HIV positive-LTB negative; HIV^+^LTB^+^: HIV-LTB co-infected; HIV^-^LTB^+^: HIV negative- LTB positive; HIV^-^LTB^-^: HIV-negative and LTB-negative. *: p < 0.05, **: p < 0.01, ***: p < 0.001.

To investigate whether HIV infection had an impact on the Th17 cytokine (IL-17A) response in LTB-infected individuals, we measured the concentration of IL-17A cytokine in plasma and from the PBMC supernatants with or without stimulation of PPD or SEB antigens. We found no significant difference in the systemic level of IL-17A in plasma between any of the study groups. Similarly, there were no significant differences in the IL-17A production by PBMCs in any of the groups (**Fig 4A and 4B**).

### HIV infection was associated with lower levels of mitogen-induced IL-10 in LTB-infected and LTB-negative individuals

**Fig 5:**
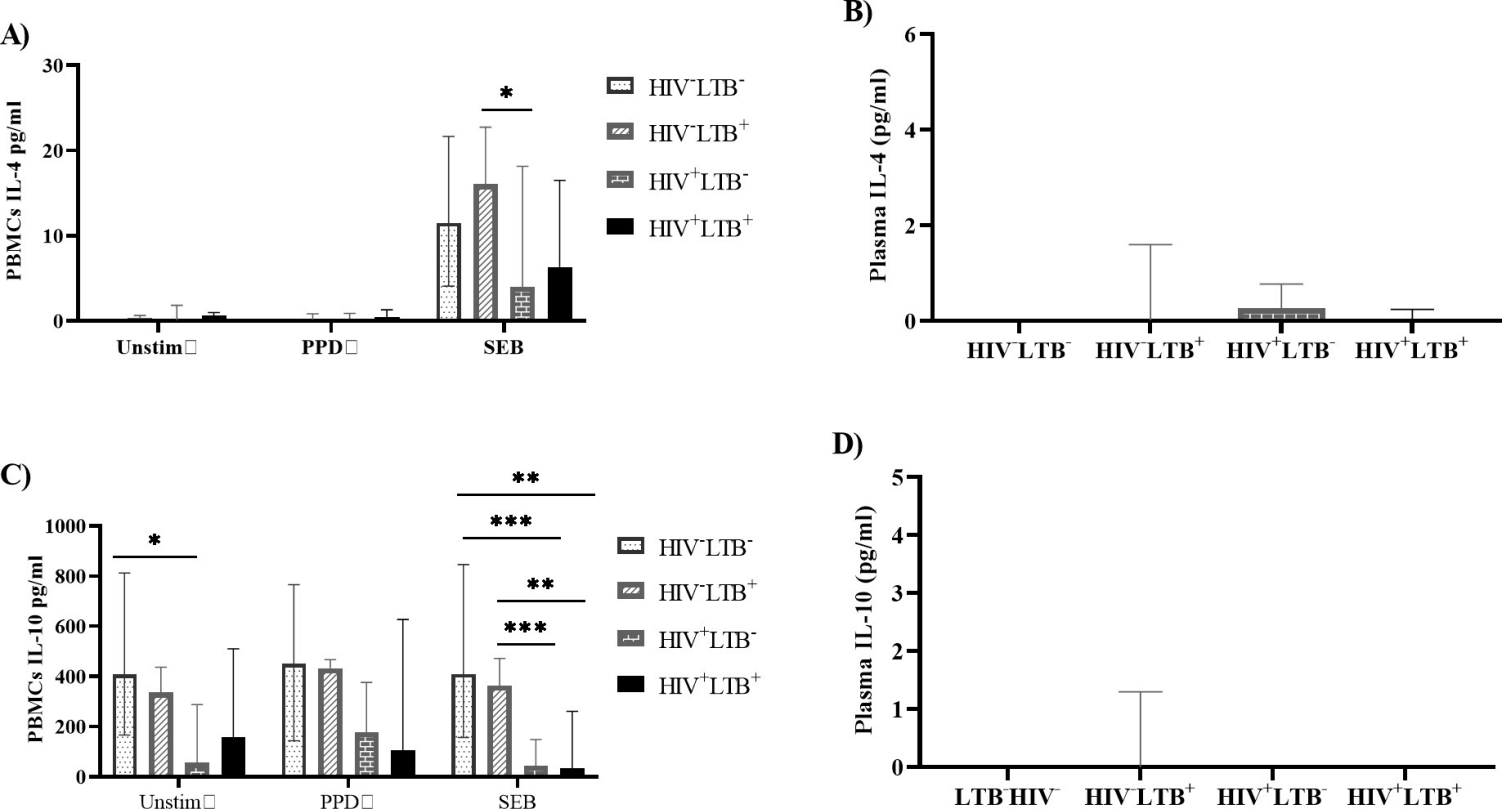
HIV infection was associated with lower levels of mitogen-induced IL-10 in LTB-infected and LTB-negative individuals. PBMCs isolated from HIV^+^LTB^-^ (n = 27), HIV^+^LTB^+^ (n = 13), HIV^-^LTB^+^ (n = 15) and HIV^-^LTB^-^ (n = 15) were stimulated with media alone (Unstim), PPD or SEB and cultured for 19 hours at 37°C, and PBMC supernatants were harvested. The levels of IL-4 and IL-10 were determined in plasma and from PBMC supernatants using a cytometric bead array. Data were presented using the median with IQR. The cytokine response was compared across the four groups using the Kruskal Wallis test, followed by Dunn’s post hoc multiple comparisons test to compare the median expression level of cytokines in each group. **A**): the level of IL4 expression by PBMCs with or without stimulation, **B**): the plasma level of L-4, **C):** the level of IL-10 production by PBMCs with or without stimulation, **D)**: the plasma level of IL-10. Unstim: unstimulated; PPD: purified protein derivative; SEB: staphylococcus enterotoxin B; HIV^+^LTB^-^: HIV positive-LTB negative; HIV^+^LTB^+^: HIV-LTB co-infected; HIV^-^LTB^+^: HIV negative-LTB positive; HIV^-^LTB^-^: HIV-negative and LTB-negative. *: p < 0.05, **: p < 0.01, and ***: p < 0.001.

We analyzed the Th2 cytokines (IL-10 and IL-4) in plasma and PBMC supernatants without stimulation and after stimulation with PPD and SEB. The systemic plasma levels of IL-10 and IL-4 were not significantly different among the study groups. Although the PPD-induced IL-10 and IL-4 production did not show any significant difference among the study groups, the baseline level of IL-10 in unstimulated PBMCs was significantly higher in HIV^-^LTB^-^ groups compared with HIV^+^LTB^-^groups (p = 0.0261). The SEB-induced IL-10 production by PBMCs was significantly lower in HIV^+^LTB^-^ (p = 0.0081) and HIV^+^LTB^+^ (p = 0.0006) groups compared with HIV^-^LTB^+^ groups. Moreover, the production of IL-4 was significantly reduced in HIV^+^LTB^-^ groups compared with HIV^-^LTB^+^ groups in SEB-stimulated PBMCs (p = 0.0276) (**Fig 5A–5D**).

## Discussion

The burden of TB and HIV substantial co-prevalence has become a public health threat around the globe [6]. Infection with HIV in LTB-infected individuals has been identified as a major risk factor for the conversion of LTB to ATB [42]. The immunological mechanisms by which HIV enhances the reactivation of LTB into ATB are not fully understood. Thus, we aimed to assess whether HIV co-infection during LTB affects the response of certain Th1, pro-inflammatory, Th17, and Th2 cytokines.

We found that HIV co-infection in LTB-infected individuals caused a significantly lowered production of PPD-induced Th1 (IFN-gamma and IL-2) cytokines by PBMCs and the systemic pro-inflammatory (TNF-alpha and IL-6) cytokines in plasma. The lower number of CD4+T cells (the subset of PBMCs and main producers of Th1 cytokines) in both HIV-positive LTB-negative and HIV-LTB co-infected individuals (**Table 1**) compared with the normal range of CD4+T cell count in healthy individuals (500–1200 CD4+ T cells/μl [43]) might explain the reduced production of Th1 cytokines in HIV-infected groups. The novel observation in our study is that there were lower levels of pro-inflammatory (TNF-alpha and IL-6) cytokines in both HIV-positive LTB-negative and HIV-LTB co-infected individuals in plasma but not in PBMCs, which might indicate that HIV infection affects other TNF-alpha and IL-6-producing cells, which are not a subset of PBMCs.

IFN-gamma activates Mtb-infected macrophages and enhances their capacity for intra-phagosomal Mtb control and the production of antimycobacterial compounds such as nitric oxides and reactive oxygen species [17, 44]. In our study, the PPD-induced level of IFN-gamma and IL-2 by PBMCs was significantly reduced in both HIV-LTB co-infected and HIV-infected LTB-negative groups compared to HIV-negative LTB-negative groups. However, there was no significant difference in IFN-gamma response between HIV-negative LTB-infected and HIV-LTB co-infected groups following stimulation of PBMCs with SEB (a mitogen to stimulate a non-specific strong T cell response), which suggests that HIV-mediated impairment of IFN-gamma production seems to be TB specific.

Our finding was in line with a previous study that demonstrated that there was a reduced CFP-10/ESAT-6 induced IFN-gamma production in HIV-LTB co-infected individuals compared to HIV-negative LTB-infected individuals [45]. It has also been shown that in HIV-LTB co-infected individuals; there was significantly reduced IFN-gamma production from whole blood culture supernatants following in vitro ESAT-6/CFP-10 stimulation compared with HIV-negative LTB-infected individuals [46]. The reduced production of IFN-gamma might partly contribute to the reactivation of LTB to ATB, as it was demonstrated by the study on the mouse model that deletion of the IFN-gamma receptor enhanced the likelihood of Mtb dissemination [47].

IL-2 promotes the survival and clonal expansion of Th1 lymphocytes to produce other essential cytokines, such as IFN-gamma, which is a potent macrophage activator [9]. We found a significantly reduced production of IL-2 in plasma and PPD-stimulated PBMCs in both HIV-LTB co-infected and LTB-negative HIV-infected groups compared with HIV-negative LTB-infected groups. Consistent with our result, it has been shown that HIV-LTB co-infected groups exhibited a reduced production of IL-2 in ESAT-6/CFP-10 stimulated PBMC supernatants compared with HIV-negative LTB-infected groups [45, 48]. Furthermore, HIV infection had caused a lower level of IL-2 cytokine response in the LTB-infected groups in ESAT-6/CFP-10 stimulated whole-blood supernatants [46].

TNF-alpha participates in regulating the formation and maintaining the structural integrity of granulomas [49]. IL-6 also plays an important role in the initiation of an early pro-inflammatory response and increasing acute-phase protein synthesis, which will favor the early control and elimination of Mtb [21]. However, in this study, we found that the systemic level of TNF-alpha and IL-6 in plasma was significantly reduced in HIV-LTB co-infected groups compared with HIV-negative LTB-infected groups. Although it is difficult to compare the cellular cytokine response in the granuloma and its secretion pattern in plasma, our finding was consistent with the previous study, which showed that lung granulomas in adults with Mtb-HIV co-infection released a significantly reduced level of TNF-alpha compared to HIV-negative Mtb-infected granulomas [50]. The previous studies related to the expression level of IL-6 during HIV and LTB co-infection are very limited. However, it has been shown that mice with IL-6 deficiency were extremely vulnerable to TB pathogenesis and eventually succumbed to it [24].

Although the primary sources of TNF-alpha and IL-6 are macrophages and peripheral blood monocytes [13, 51], we found that the production of these cytokines is significantly impaired in plasma but not in the PBMCs in HIV-LTB co-infected groups. Other innate immune cells such as mast cells, dendritic cells, and eosinophils and non-immune cells like epithelial cells, endothelial cells, and fibroblasts are secondary sources of these cytokines during acute inflammatory conditions [52, 53]. The significantly reduced production of these pro-inflammatory cytokines in plasma but not in PBMCs during HIV-LTB co-infection might indicate that HIV infection impairs the production of these cytokines in the effector cells other than PBMCs.

During LTBI, the immune response is mainly cell-mediated and driven through the release of cytokines such as IFN-gamma and TNF-alpha, which enhances the polarization of macrophages to a classically activated (M1) type, having high Mtb killing capacity and exhibiting higher production of antimicrobial substances such as reactive oxygen species and nitric oxide [54, 55]. Moreover, IFN-gamma and TNF-alpha are involved in the formation of granuloma, in which the Mtb could be contained [10, 20]. IL-6 also plays an important role in the initiation of an early pro-inflammatory response against intracellular Mtb [21]. In general, the observed impaired production of Th1 cytokines (IFN-gamma and IL-2) and pro-inflammatory cytokines (TNF-alpha and IL-6) during HIV co-infection in LTB-infected individuals might favor intracellular Mtb growth and fail to control the conversion of LTBI into ATB.

Increased levels of IL-4 and IL-10 can affect Mtb-induced granuloma and are linked to an increased bacterial burden, and more severe TB and LTBI recurrence [13, 56]. However, our result showed that HIV infection in LTB-infected or LTB-negative individuals did not significantly affect the production of either IL-10 or IL-4.

Our small sample size might be considered as a limitation of the study. In addition, we could not try to see the impact of HIV infection on the cytokine responses of LTB-infected individuals in each HIV clinical stage due to the limited number of participants in each clinical stage.

## Conclusion

In general, our study demonstrated that there was a reduced expression of Th1 cytokines such as IFN-gamma and IL-2 in PPD-stimulated PBMCs in HIV-LTB co-infected and HIV positive-LTB negative individuals. The systemic plasma levels of IL-2 and pro-inflammatory cytokines (TNF-alpha and IL-6) were also lower in both HIV positive-LTB negative and HIV-LTB co-infected groups compared with HIV negative-LTB infected groups. Our findings indicated that HIV infection influences the production of cytokines in individuals with LTBI, which may contribute to the conversion of LTBI into active TB.

## Supporting information

S1 Table Correlation analysis of age and BMI with cytokine levels. (DOCX)

## Abbreviations

ART: Antiretroviral therapy
ATB: Active tuberculosis
CBA: Cytometric bead array
CFP-10: Culture filtrated protein-10
ESAT-6: Early antigenic target-6
HIV: Human immunodeficiency virus
IFN-gamma: Interferon-gamma
LTBI: Latent tuberculosis infection
Mtb: *Mycobacterium tuberculosis*
PBMCs: Peripheral blood mononuclear cells
PE: Phycoerythrin
PPD: Purified protein derivative
QFT plus: QuantiFERON-TB Gold plus
SEB: Staphylococcal Enterotoxin B
SIV: Simian immuno-deficiency virus
TB: Tuberculosis
TNF-alpha: Tumor necrosis factor-alpha
UoG-CSH: University of Gondar Comprehensive Specialized Hospital
WHO: World Health Organization
