# Supplementary material for: Human-immunodeficiency virus infection associated with the impaired Th1 and pro-inflammatory cytokine response in latent tuberculosis-infected individuals: A comparative cross-sectional study
Source: PLoS One. 2024 Nov 8;19(11):e0313306. doi: 10.1371/journal.pone.0313306 (PMC11548834; doi:10.1371/journal.pone.0313306)
Supplement: S1 Table — (DOCX) [file pone.0313306.s001.docx]

| S1 Table: Spearman’s correlation analysis of age and BMI with each cytokine levels. | | | | | | | | |
| --- | --- | --- | --- | --- | --- | --- | --- | --- |
|  | HIV^+^LTB^-^ | | **HIV^+^LTB^+^** | | **HIV^-^LTB^+^** | | **HIV^-^LTB^-^** | |
| Cytokines | **Age: r (p)** | **BMI r (p)** | **Age r (p)** | **BMI r (p)** | **Age r (p)** | **BMI r (p)** | **Age r (p)** | **BMI r (p)** |
| Plasma IL-2 | NR | NR | NR | NR | -0.294 (0.282) | -0.356 (0.190) | 0.053 (0.850) | -0.050 (0.862) |
| Unstim IL-2 | 0.151 (0.451) | -0.025 (0.900) | NR | NR | 0.129 (0.642) | 0.124 (0.657) | 0.178 (0.527) | 0.087 (0.761) |
| PPD IL-2 | NR | NR | 0.232 (0.538) | 0.000 (>0.999) | 0.244 (0.377) | 0.064 (0.822) | 0.325 (0.234) | -0.015 (0.959) |
| SEB IL-2 | -0.077 (0.702) | 0.508 (0.006)* | -0.326 (0.273) | 0.167 (0.581) | 0.520 (0.048)* | 0.521 (0.048)* | 0.203 (0.463) | -0.057 (0.839) |
| Plasma IFN-gamma | -0.147 (0.464) | -0.011 (0.953) | -0.038 (>0.999) | 0.308 (0.461) | -0.331 (0.229) | -0.142 (0.617) | 0.309 (0.267) | 0.071 (0.805) |
| Unstim IFN-gamma | -0.144 (0.471) | 0.102 (0.609) | -0.224 (0.456) | 0.390 (0.188) | -0.101 (0.714) | 0.023 (0.936) | -0.025 (0.930) | -0.265 (0.335) |
| PPD IFN-gamma | -0.059 (0.769) | 0.045 (0.820) | -0.227 (0.450) | 0.259 (0.930) | -0.256 (0.352) | -0.253 (0.360) | -0.499 (0.094) | -0.273 (0.321) |
| SEB IFN-gamma | -0.033 (0.866) | 0.174 (0.383) | -0.320 (0.283) | 0.274 (0.363) | 0.048 (0.864) | 0.135 (0.629) | -0.203 (0.463) | -0.135 (0.626) |
| Plasma TNF-alpha | 0.197 (0.324) | 0.178 (0.373) | 0.360 (0.225) | 0.156 (0.616) | -0.293 (0.284) | -0.384 (0.157) | 0.040 (0.887) | -0.052 (0.854) |
| Unstim TNF- alpha | 0.047 (0.813) | -0.140 (0.485) | -0.486 (0.093) | 0.090 (0.767) | 0.019 (0.946) | 0.175 (0.532) | 0.016 (0.956) | -0.305 (0.266) |
| PPD TNF- alpha | 0.108 (0.590) | -0.082 (0.683) | -0.580 (0.040)* | 0.175 (0.565) | 0.084 (0.764) | 0.171 (0.540) | 0.032 (0.909) | -0.370 (0.174) |
| SEB TNF- alpha | 0.051 (0.799) | 0.190 (0.340) | -0.663 (0.015)* | -0.164 (0.591) | 0.280 (0.309) | 0.192 (0.490) | 0.216 (0.435) | -0.085 (0.760) |
| Plasma IL-6 | 0.150 (0.453) | 0.015 (0.939) | 0.411 (0.163) | -0.020 (0.956) | -0.441 (0.100) | -0.440 (0.101) | 0.095 (0.732) | -0.040 (0.888) |
| Unstim IL-6 | -0.184 (0.355) | 0.137 (0.495) | -0.596 (0.034)* | 0.005 (0.992) | 0.278 (0.312) | 0.335 (0.221) | -0.041 (0.884) | -0.110 (0.692) |
| PPD IL-6 | -0.101 (0.615) | 0.226 (0.256) | -0.569 (0.045)* | -0.192 (0.529) | 0.195 (0.481) | 0.167 (0.549) | 0.238 (0.389) | -0.087 (0.755) |
| SEB IL-6 | 0.023 (0.906) | 0.328 (0.094) | -0.555 (0.051) | -0.280 (0.353) | 0.438 (0.103) | 0.314 (0.253) | 0.027 (0.925) | -0.039 (0.890) |
| Plasma IL-17A | -0.113 (0.573) | -0.252 (0.204) | -0.038 (>0.999) | 0.308 (0.461) | -0.186 (0.666) | -0.371 (0.266) | 0.078 (0.782) | -0.270 (0.330) |
| Unstim IL-17A | -0.084 (0.675) | 0.160 (0.423) | -0.345 (0.245) | 0.418 (0.156) | -0.121 (0.664) | 0.451 (0.093) | -0.004 (0.990) | -0.078 (0.778) |
| PPD IL-17A | 0.027 (0.891) | 0.260 (0.190) | -.0351 (0.235) | 0.253 (0.399) | -0.444 (0.097) | -0.474 (0.076) | -0.122 (0.663) | -0.358 (0.188) |
| SEB IL-17A | -0.014 (0.944) | 0.563 (0.002)* | -0.437 (0.135) | 0.145 (0.632) | -0.217 (0.433) | -0.317 (0.248) | 0.374 (0.168) | -0.218 (0.429) |
| Plasma IL-4 | -0.295 (0.134) | 0.042 (0.833) | -0.083 (0.782) | -0.050 (0.873) | 0.086 (0.757) | 0.444 (0.098) | 0.350 (0.223) | 0.102 (0.728) |
| Unstim IL-4 | 0.298 (0.130) | 0.204 (0.307) | 0.011 (0.971) | 0.390 (0.186) | 0.384 (0.156) | 0.201 (0.467) | -0.064 (0.800) | 0.102 (0.728) |
| PPD IL-4 | -0.007 (0.971) | 0.152 (0.446) | -0.186 (0.539) | -0.243 (0.422) | -0.320 (0.242) | -0.130 (0.641) | 0.243 (0.642) | 0.103 (0.857) |
| SEB IL-4 | 0.255 (0.198) | 0.409 (0.034)* | 0.320 (0.281) | 0.475 (0.102) | -0.269 (0.329) | -0.178 (0.523) | 0.501 (0.058) | 0.098 (0.726) |
| Plasma IL-10 | NR | NR | NR | NR | -0.297 (0.278) | -0.092 (0.740) | 0.381 (0.176) | 0.078 (0.781) |
| Unstim IL-10 | -0.250 (0.208) | 0.158 (0.428) | -0.530 (0.065) | 0.099 (0.749) | 0.089 (0.749) | -0.157 (0.575) | 0.043 (0.879) | 0.014 (0.961) |
| PPD IL-10 | -0.264 (0.183) | 0.196 (0.327) | -0.505 (0.080) | 0.132 (0.667) | 0.201 (0.468) | -0.150 (0.590) | 0.272 (0.323) | 0.080 (0.775) |
| SEB IL-10 | -0.281 (0.155) | 0.406 (0.035)* | -0.501 (0.082) | 0.142 (0.640) | 0.484 (0.068) | 0.139 (0.620) | 0.164 (0.556) | 0.284 (0.302) |

*: significant correlation using spearman’s correlation analysis, r: correlation coefficient (where; 0: no correlation, < 0.3: weak, 0.3-0.7: moderate, and > 0.7: strong association), p: p-value, NR: not reported, HIV^+^LTB^-^: HIV positive LTB negative, HIV^+^LTB^+^, HIV positive and LTB positive, HIV^-^LTB^+^: HIV negative LTB positive, HIV^-^LTB^-^: HIV negative and LTB negative, Unstim: unstimulated, PPD; purified protein derivative, SEB; staphylococcus enterotoxin B, IL-2: interleukin 2, IFN-gamma: interferon gamma, TNF-alpha: tumor necrosis factor alpha, IL-6: interleukin 6, IL-17A: interleukin 17A, IL-4: interleukin 4, IL-10: interleukin 10.
